# The experience of an innovative interdisciplinary model of primary care delivery in changing organizational dynamics: a grounded theory study

**DOI:** 10.1017/S1463423625000210

**Published:** 2025-02-28

**Authors:** Elisabetta Mezzalira, Jessica Longhini, Elisa Ambrosi, Giulia Marini, Luisa Saiani, Achille Di Falco, Chiara Leardini, Federica Canzan

**Affiliations:** 1 Department of Biomedicine and Prevention, University of Rome Tor Vergata, Rome, Italy; 2 Department of Diagnostics and Public Health, University of Verona, Verona, Italy; 3 Azienda ULSS 8 Berica, Regione del Veneto, Vicenza, Italy; 4 Department of Business Administration, University of Verona, Verona, Italy

## Abstract

**Introduction:**

Changing dynamics are pushing institutions to focus on care delivery innovation. To address the shortage of general practitioners (GPs), an Italian health district recently introduced a new primary care model called Primary Health Point (PHP) to provide primary integrated care to its population.

**Aim:**

To investigate the healthcare professionals’ (HCPs) experience regarding the introduction of the PHP and to describe its process of care delivery.

**Methods:**

Qualitative study design with a grounded theory approach and convenience sampling. Interviews were conducted using a semi structured guide to explore the experience of HCPs working at the PHP. The development of open coding was followed by the creation of categories. The analysis was conducted utilizing NVivo software.

**Results:**

Twelve HCPs working at the PHP were interviewed and highlighted the model structure. The themes were *the context and the antecedents* that identified the most common health complaints and the patients with more needs and reflected on the traditional GP model; *the process,* which highlighted the complexity of interdisciplinary teamwork and the role of the Family and Community Nurse (FCN) in the new model; *the outcome* identified the factors mediating satisfaction with the care delivered by the PHP.

**Conclusions:**

The PHP has been considered a possible alternative to the GP model by its end users. It addresses disease pathway coordination, referrals, and medication management, focusing on chronic and older adult populations. It features interdisciplinary workflows with rotating physicians and consistent family nurse support. Proactive monitoring and a focus on disease education benefit fragile patients.

## Introduction

The World Health Organization (WHO) has recently reaffirmed the importance of ‘primary health care’ (PHC), as the most inclusive, effective, and efficient approach to enhance people’s physical and mental health, as well as social well-being (WHO, 2018). ‘Primary care’ is defined by the WHO as a model of care that supports first-contact, accessible, continuous, comprehensive, and coordinated person-focused care. It aims to optimize population health and reduce disparities across the population by ensuring that subgroups have equal access to services (WHO, 2024). Strong primary care, defined as coordinated, comprehensive first-contact care, is indeed associated with a better functioning health system, leading to positive effects such as mortality reduction, improved population health outcomes, and lower healthcare costs (Reader, 1993; Engström *et al*., 2001; Kringos *et al*., 2013; Wensing *et al*., 2019; Sawicki *et al*., 2021). It is also associated with a lower risk of hospitalization in high-risk patients (Sawicki *et al*., 2021), and it represents the only feasible pathway to ensure healthy ageing and high quality of life to users in the context of phenomena such as multimorbidity and the ageing population (van Weel and Kidd, 2018).

Nowadays, healthcare organizations are asked to answer to the increased demand for acute healthcare services and specialty care and at the same time strengthen primary care services delivery (Hunter and Bengoa, 2022). Italian and European healthcare providers are facing an increase in care complexity due to ageing population with multiple chronic illnesses and changing expectations from the population. However, a variety of factors, such as general practitioners (GP)’ shortages and lack of systematic integration among providers (Auschra, 2018; Endalamaw *et al*., 2023), make strengthening a traditional primary care model increasingly difficult. For these reasons, different government and institutions have focused their efforts on the innovation of their models of care, to address the complex needs of users, as highlighted by a 2023 umbrella review on innovative models of care, covering evidence from 66 reviews and more of 1000 articles (Roberts *et al*., 2023).

In 2014, the National Health Service in England launched a new strategy to better coordinate care across primary care, community services, and hospitals. As part of this, they initiated a new care models programme where a total of 50 local pilot sites were selected to develop and test five different types of integrated care models with support from a national team (Starling, 2018). The new care models tested included connecting care homes into the healthcare system, integrated providers of out-of-hospital care, integrating hospital, primary, community, and mental health services, urgent and emergency care models, and acute care collaboration models between hospitals. These models aimed to improve population health, patient experience, and reduce costs by better coordinating services for groups like the elderly, those with chronic conditions, and high-risk patients.

A model of care can entail the organization of care in the healthcare system or more specifically a care delivery system at a ward or unit level (Geltmeyer *et al*., 2024). The creation process of a new care model entails understanding the problem and the needs of end users, designing a solution, implementing the change, and monitoring its long-term sustainability (Kathryn *et al*., 2021; Jarrod *et al*., 2023). New models of care are emerging in the context of primary care (Roberts *et al*., 2023). Specifically, there have been efforts in European primary care settings to innovate primary care delivery through the introduction of new models, digitalization and population stratification initiatives, integration strategies, as well as through the development of new figures, such as care and case managers and Family and Community Nurses (FCN) (Hudon *et al*., 2019; Conti *et al*., 2021; Wong *et al*., 2022; Mezzalira *et al*., 2024).

Following these recent European trends of model innovation, to address the challenge of the critical shortage of GPs in the area, in December 2021 a Local Heath Authority situated in the Veneto Region introduced a new primary care model, from here on defined as ‘Primary Health Point’ (PHP). The decentralized nature of Italy’s healthcare system allows regional authorities to independently organize and pilot new delivery models tailored to local needs. In the Veneto region specifically, the local health authority identified a shortage of GPs to provide primary care services to the local population. With the autonomy to introduce solutions to address this gap, the region introduced the PHP in a pilot site as an alternative interdisciplinary team-based model integrating FCNs, rotating physicians, and extended service hours. This pilot innovative primary care approach could be extended to other local health authorities in Veneto and other Italian regions facing the same issues.

This model, which strives to deliver care with an integrated approach, aims to provide an alternative solution in absence of an adequate number of GPs caring for the local population and improve access to primary care services for citizens. The PHP represents an evolution of the traditional GP-centric primary care model; however, it remains unclear whether it might constitute an appropriate alternative to it. As highlighted by different experts (Donetto *et al*., 2015; Bird *et al*., 2020, 2021) HCPs, which are the final end-users of the service, along with patients, need to be involved in the co-design and analysis of the care process they contribute to deliver, as they are the actors best suited to properly highlight strengths and weaknesses of the novel model to make improvements. For this reason, the aim of this study was to investigate the experience of HCPs regarding the introduction of the new primary care model and to describe the process of delivering care within the new care model, using an experience-based co-design approach.

## Methods

### Design

We used a qualitative case study design with a constructivist grounded theory approach to explore the experience of HCPs working in an innovative integrated primary care model. Constructivist grounded theory focuses on how participants construct meanings and actions, aiming to develop an interpretive understanding grounded in the data (Charmaz, 2006). This approach was well suited to examine HCPs’ subjective experiences and derive a theoretical explanation of the process of care delivery in the new model (Mills *et al*., 2006).

The study followed the principles of inductive iteration, comparative analysis, and abductive reasoning central to constructivist grounded theory (Chun Tie *et al*., 2019). Consistent with this methodology, we employed strategies such as initial coding, focused coding, theoretical sampling, memoing, and constant comparison throughout the data collection and analysis process (Charmaz, 2006; Sbaraini *et al*., 2011).

### Setting

The new PHP comprises an interdisciplinary team with FCNs, as well as out-of-hours service physicians and structured administrative support (Reeves *et al*., 2018). This team-based approach allows for a variety of HCPs to collaborate in delivering care. With the new model, citizens are assigned to the PHP rather than individual GPs. Depending on the urgency of their needs, they may be cared for by one of the FCNs or different physicians present that specific day on shift. The novelty of the model is given not only by the lack of patient assignment to a specific physician but also by the introduction of the family and community nurses, which are a novel figure in the Italian nursing landscape, with functions which still vary greatly from region to region based on local needs and interpretation of the general profile description. The FCNs hired by the PHP are registered nurses, which held a Master of Science in Family and Community Nursing as well as had previous experience in home care services. In the context of the PHP, these nurses have been tasked to identify and apply a proactive medicine approach in the care of chronic patients, working autonomously as well as in an interdisciplinary manner. The PHP team is composed of twelve out-of-hours service physicians, two FCNs and an additional physician available for phone call consultations. The physicians employed by the PHP are not specialized in general practice (they are either GP in training or awaiting for placement in their chosen specialty) but are available to provide care during specified hours, following a shift model and are self-employed.

The PHP operates from 8 am to 8 pm, providing extended hours compared to traditional GP practices. Calls are filtered by trained lay administrative staff. Based on the severity of the complaint, FCNs decide if to address the complaint by offering telephone consultation or to schedule a face-to-face appointment with an RN or a physician either in the same day or over the following days. This extended availability aims to accommodate the diverse schedules and needs of citizens seeking healthcare services. The PHP is financially supported by the national healthcare system. If a new GP practice starts its service in the local community, users have the choice to remain with the PHP or opt out and be assigned to the newly available GP.

## Study participants

The study followed a convenience sampling approach. Following approval from the Regional Institutional Ethical Committee, the Local Health Authority provided the researchers with a list of HCPs fitting the inclusion criteria.

For nurses, the inclusion criteria were the two FCNs employed at the PHP since its inception in December 2021, as they had been involved from the start in developing and implementing this innovative interdisciplinary model.

For physicians, the criteria were having at least 1 month of work experience at the PHP, given their rotating schedule of shifts at this facility. This 1-month minimum ensured the physicians had sufficient exposure to the interdisciplinary workflows and processes of the PHP model.

By including the two pioneering FCNs along with physicians who had worked at the PHP for at least 1 month, the researchers could capture perspectives spanning different levels of familiarity and experience with operationalizing this novel interdisciplinary approach to primary care delivery.

Patient and Public Involvement (PPI)

This study employed an experience-based co-design approach, which actively involves users in the design and conduct of research (Donetto *et al*., 2015). At the beginning of the project, the researchers consulted with all end users to gather their point of view on the PHP. These insights informed the initial development of the interview guide and study focus that was shared with PPI representatives before to start with the data collection process.

## Data collection

At the beginning of the interview meeting, the researchers presented themselves and the goals of the project, specifically a detailed description of the research project and objectives was given and informed consent was collected. No other people were present at the scheduled meetings other than the participants and the researchers. The location chosen for the interview varied based on participants’ preferences and needs: interviews were conducted either at the PHP, where a private room had been dedicated to this task in order to maintain participants’ privacy, or at the GP practices currently employing the participants. There was no prior relationship between the interviewers and the study participants. The interviews were performed by four trained and experienced female researchers, specifically three PhD nurse researchers (JL, Research Fellow in Nursing Science; EA and FC Associate Professors in Nursing Science) and a PhD Candidate in Nursing and Public Health (EM). EA and FC had extensive knowledge and teaching experience in qualitative research, which was also the methodology of their PhD dissertation thesis. JL had experience and expertise in mixed methods research and qualitative research gained during her PhD, while EM had the opportunity to deepen her training in qualitative interviewing in the course of her ongoing PhD.

The study followed a convenience sampling approach. The interviews were guided by a semi-structured guide built following the principles of the experience-based co-design approach (Supplementary 1). This approach aligns with constructivist grounded theory by facilitating the active participation of participants in the co-construction of knowledge. Consistent with a constructivist grounded theory methodology (Charmaz, 2006), the data collection and analysis occurred as an iterative process for the participants enrolled through convenience sampling. The first two interviews served as a pilot to guide refinements to the initial semi-structured guide based on participants’ responses and the researchers’ experiences during those interviews. Data from the pilot interviews were included in the full analysis. After the pilot, the semi-structured guide was further revised through the constant comparative process central to grounded theory (Charmaz, 2006). As data collection and analysis progressed concurrently, the guide was modified to explore emerging concepts and categories identified during coding and memoing activities. This allowed the researchers to progressively focus on relevant data.

The interviews had an average length of 20 minutes, ranging from 15 to 45 minutes. Each interview was audio recorded and faithfully transcribed. In line with the principles of pseudo-anonymization, an ID was assigned to each participant’s interview. Word files containing the transcripts and other materials such as memos, field notes, and the researcher’s journal were kept in a password-protected computer accessible only to researchers.

### Rigour and trustworthiness

The trustworthiness of the study was pursued using strategies outlined by Lincoln and Guba (1985). Reflexivity was practiced by journaling pre-existing knowledge, ideas, and assumptions prior to data collection. Four researchers with nursing backgrounds performed the interviews; they had not previously met the participants.

Data triangulation was achieved by analyzing and converging evidence from multiple data sources – the interview transcripts as well as documents like memos, field notes, and the researcher’s journal. The analyses were done independently by two researchers experienced in qualitative research (EM, JL) and supported by two researchers (EA, FC) expert in grounded theory to ensure multiple perspectives. Disagreements were resolved through discussion or consultation with methodological experts, who also reviewed the trustworthiness of categories and theory development.

Trustworthiness was further upheld through adhering to grounded theory methodology tenets like constant comparative analysis, thorough memoing, coding to achieve theoretical saturation, and triangulation of the various data sources (Charmaz, 2006). Maintaining an auditable trail of analytical decisions and interpretations also supported confirmability.

### Data analysis

We engaged in open, focused, and theoretical coding following a constructivist grounded theory approach (Charmaz, 2006). Open coding was collaboratively developed by the four researchers based on the initial interviews, wherein units of meaning were identified. Subsequently, two researchers applied focused coding to the remaining interviews to synthesize and categorize the initial codes. Memoing tracked and elaborated the categorization of coded data. As the analysis progressed, we engaged in theoretical sampling by pursuing relevant data to develop the properties of the emerging categories and theory. The analysis was conducted utilizing NVivo software (QSR International®, version 11). The results are reported in accordance with the COREQ (COnsolidated criteria for REporting Qualitative research) Checklist (Tong *et al*., 2007).

## Results

A total of twelve participants, two nurses and ten physicians, accepted to participate in the study and to describe their experience with the care process and primary care model. The age range of participants varied from 27 to 40 years, eight males and four females. At the time of the interviews, some physicians were still working at the PHP, while others had accepted placements as GPs in the local community after working at the PHP since its conception in December 2021. They were substituted by other physicians who had just started with one to two months of experience at the PHP. As regarding the number of nurses interviewed, it is important to note that the PHP service had only two family and community nurses employed at the time this innovative model was introduced in December 2021. As these were pioneering roles with advanced training requirements, there were no additional nurses available to recruit for this qualitative study.

**Fig. 1.**
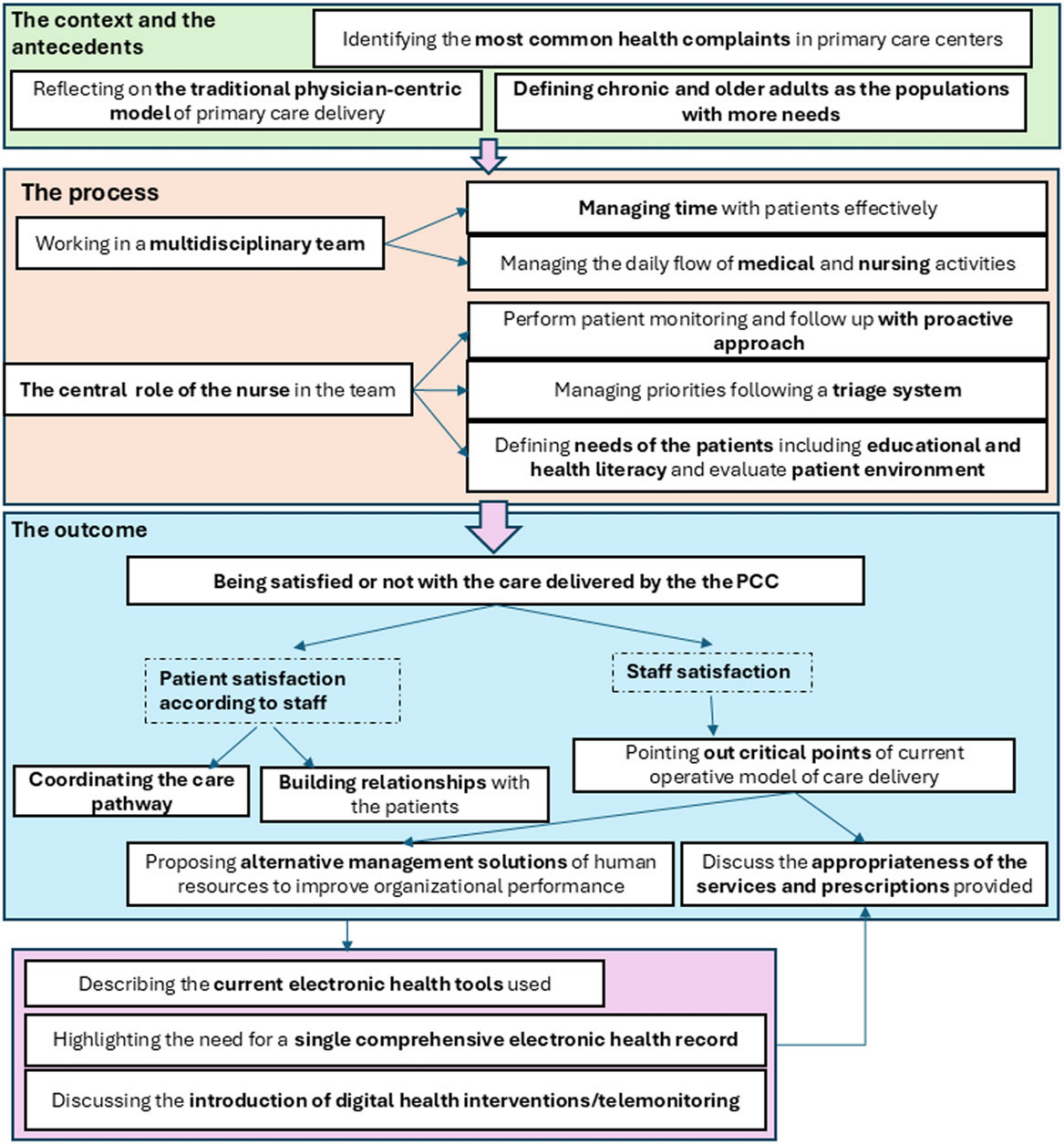
The process of the PHP.

The substantive theory that emerged from this study was an innovative model of primary care delivery in changing organizational dynamics. Figure 1 displays the context and antecedents, process, and outcome of the theory. Each of these elements, along with the categories that constitute each element, is described sequentially below.

### Theme: the context and the antecedents

1. **1.1 During a typical shift at the primary care center, physicians commonly handle tasks such as prescribing medications, reviewing diagnostic results, and consulting patients on symptoms like back pain or chronic conditions such as arterial hypertension**.

*So many times, they come for consultation regarding a specialist’s report, let’s say interpretations are quite frequent. Then problems with blood pressure, low back pain, hernias--then re-prescription requests for chronic medications. (I4)*

Some users come to the PHP after many years without a visit with a GP and ask for the renewal of old prescriptions for medications that they don’t need any more or that are not appropriate in their case.

1. **1.2 Reflecting on the traditional physician-centric model of primary care delivery**

Physicians working at the PHP perceive the tendency of users to ask for the same doctor at the PHP again and again. Chronic, complex, and elderly users seem to prefer to maintain a single physician in charge of their pathway, to guide them throughout the different steps.

*I think it was also felt by the users because anyway there was sometimes a tendency to ask for the possibility of referring to the same doctor, the one who had already followed them, so I think it was also a little bit the need of the patient.(I 7)*

*Perhaps the patient is somewhat missing the GP as the main figure of reference, especially in the elderly, is the most important thing for them.* (I10)

However, according to HCPs, users emphasize the importance of continuity of care with a consistent health professional, whether it be a nurse or a doctor, rather than specifically requiring a physician as their point of reference. The focus is on having a consistent healthcare provider to ensure continuity of care.

*Sometimes they lack a figure who is always the same, who is well organized and who tells them what to do. (*I12*)*

Moreover, some users prioritize efficiency over establishing a personal relationship with a specific doctor. The impact of the PHP on patient experience depends on patient expectations, whether they prefer continuity and human connection or efficiency. According to some HCPs interviewed, younger adults tend to prefer a primary health system that responds promptly, while older individuals prefer a single point of reference despite potential wait times.

*The elder wants continuity, the younger wants service. The younger person prefers fast performance, while the older person is willing to wait a couple of days to get things done right.* (I6*)*

It has also been pointed out that not having assigned a specific pool of users can help doctors to maintain a freer and more professional relationship with them. Having that relationship that is established between GP and patient can create the conditions for the patient to demand a little bit more.

*The patient demands everything to be done right away because of the direct relationship with the HCP, he/she expects to be seen without an appointment etc., so the patient takes a little bit more freedom. Whereas by being visited by different physicians all the time there is more detachment, they are more respectful of the figure of the doctor. (I10)*

1. **1.3 Defining chronic and older adults as the populations with more needs**

The patient populations coming to the PHP with more healthcare and social needs to be met, and therefore presenting the highest care load for the service, according to the HCPs, were older adults (specifically over 70–75 years old), bedridden patients, psychiatrically ill, or patients who have not had a GP appointment for a long time or had been poorly attended by the previous GP.

*Certainly, the elderly, over 70*–*75 years old.* (I 11)

*Either there were those who came because they thought they had needs but really just needed to be reassured that everything was okay-or there were those who had a deteriorated health status for some reason. And it wasn’t just those with poor health, maybe you would do the tests on patients feeling well and see that they had sky-high cholesterol, or they had had an acute episode and had gone to the emergency department. In some cases, they had to be completely reassessed with respect to management of their diseases. (*I 8)

Chronic and frail patients (e.g. diabetes, hypertension, rheumatology), with polypharmacotherapy, INR monitoring, were identified as those with more frequent access, due to the need for frequent monitoring and follow-up visits.

Moreover, the HCPs recognized in older adults and social cases the most challenging situations to manage, in particular, frail older adults with chronic conditions were considered as particularly in need of care, whether they lived alone or had a partner. Indeed, in the case of couples, very often both of the partners presented health needs which required to be managed simultaneously.

*The frail, chronic elderly man, alone or not always alone, maybe with the wife, another frail, chronic elderly. Sometimes it happens that we focus on one of them, you manage the husband and then the wife pops up telling you “you know since yesterday I have high blood pressure…” and from there the second pandora’s box opens. (I1)*

### Theme: the process

**2.1 Working in a interdisciplinary team**

Physicians and nurses seemed to appreciate the opportunity to work in a team to deliver primary care, compared to the traditional GP practice. Their perception is that the interdisciplinary approach improves response to needs and efficiency of service use, improving global patient care. Physicians appreciated the opportunity to engage with other physicians to solve the patient’s problem noting that working in groups (at the PHP) allows for more support and more exchange of ideas with respect to integrated medicine/GP modalities. Furthermore, all the team members recognized the value of providing time for interdisciplinary discussion of the most complex cases. Moreover, nurses highlighted that the development of an integrated care model is a work in progress, building trust, and partnerships among members.

*The combination of outpatient medical activity, remote medical activity, and nursing clinic is considered a winning choice for providing a fair, if not excellent, quality of care (*I3).

Working in team can also prove to be challenging, with proactive, interdisciplinary patient care hindered in some cases by physician-nurse communication difficulties (nurse is not alerted regarding users deserving follow-up).

*We collaborate with all physicians, however, not all of them have our proactive view. At the end of the shift maybe you ask if there are any users to refer to, the physician says no. Then maybe that patient comes back after two weeks, you read the handoff and notice that that patient was worthy of an intermediate follow up call. Or, for example, you highlight users in yellow* [as users of the list to be visited together] *and then maybe you don’t get called by the doctor because maybe he’s in a hurry. It doesn’t happen to everybody, but sometimes that happens. (I2)*

**2.2 Managing the daily flow of medical and nursing activities.**

The HCPs perceive it as important and positive to give service users the opportunity to have an 8am–8pm service. *Maybe there will be some lack of continuity, however the possibility anyway to have an 8*–*20 service is important. These are extra services that you get by not having the same reference person all the time, I can’t tell you if that is a pro or a con (I 6)*

**2.3 The role of the nurse in the team and in ensuring continuity of care**

FCNs run their clinic independently from physicians, providing not only advanced dressings or vaccination services, they perform also in-person visits with monitoring purposes and follow-up calls, home visits alone, or in combination with physicians. Specifically, home visits are predominantly nurse-led even for acute clinical assessments. Nurses at the PHP have also taken on the management process of aids prescription, prescribing aids and necessary devices after home assessment, as well as the process for the disability claim activation request.

*There I proposed the activation of the disability application, the request for aids with an equipped bed, the request for side rails, the basic anti-decubitus mattress. We uncovered the patient, did the parameters, examined her from head to toe, also asked for diapers. I saw that she had incontinence aids, I saw them on the couch on a box, to my request ‘did you requested the diapers for your mom to the healthcare services?’ she replied ‘these were provided to me by a neighbor whose dad died, they were leftover and she gave them to me’. So we also requested incontinence aids. (I1)*

*Interprofessionality and the presence of nurses are an added value because nurses are fixed presences and have the opportunity to get to know fragile patients more in-depth and perform monitoring (I 12)*

Physicians increasingly recognize the value of nurses’ work, considering stimulating them to work with nurses who do preventive medicine and noting that users at the PHP receive more services because of the presence of FCNs compared to traditional general practices, for example, it enables a less frequent activation of home services. Moreover, FCNs embrace the opportunity of caring and managing the patient with this holistic perspective, although there are challenges related to the novelty of the model and the new profile of family nurses, which are developing their profile, tasks, and functions compared to traditional family nurses.

**2.4 Performing patient monitoring and follow-up with proactive approach**

The presence of the FCN is an advantage to the team because he or she applies a proactive medicine approach, identifying with the equipe a list of frail users in need of closely monitored, assessing the adherence to exams and prescriptions and their effectiveness.

*Having a list of frail users and knowing all of them allows you to call and ask a little bit how it is going, or for example there were also schedules. We knew that the patient had to have a cardiology visit for decompensation in those months there, the nurse would call him and ask “Have you had the visit? The patient would answer negatively and then she would say come here and we will schedule your visit. He was more proactive (I5).*

***2.5* Defining needs of the users**

Nurses highlighted the perception of large gaps in patient knowledge regarding treatment and the need for accurate therapeutic education by professionals, for example, regarding education on correct blood pressure measurement.

*Certainly, practical needs as well, like measuring blood pressure and parameters. At home maybe they don’t have the device or maybe they have it but very old and therefore not reliable. So many times, we have the device brought in and we do an additional live check because we often find discordant values (I2)*

FCNs conduct advanced assessments of users’ educational and social-health needs, uncovering hidden needs and facilitating access to social services when necessary. They also provide therapeutic education during home visits, educating caregivers on preventing pressure ulcers and ensuring appropriate patient mobilization. At the PHP, they assist older users in understanding service dynamics and navigating the differences between PHP and home care services effectively.

*the whole aspect of even social support sometimes. You go a little bit in depth, asking about how is your home structured? do you live alone? But can you make your own food? Who is coming to help you?” we use these questions to find out whether it is necessary to activate the social services or home delivered meals or offer certain aids. Sometimes they come here for one reason but then you discover so many other needs. By dint of seeing them you also get to know them. What is discovered is never the reason that is stated. There are so many unexpressed needs. (I1)*

**2.6 Managing priorities following a triage system**

Nurses prioritize team tasks daily, focusing on urgent visits, home visits, unseen users from the previous day, and medical certificate needs. They also monitor daily hospital discharge notifications and hospitalizations, contributing to the system’s overall efficiency compared to traditional GP practices.

*in the end they then have the immediate response, if you think about it, they call the call center and within 2 3 days they already have the answer… with the family doctor sometimes unfortunately it happens to wait even a few days, instead, with this service everything considered we also manage to have a perfect timing, because in the end every urgent case get to be seen immediately. (I1)*

### Theme: the outcome

Patient satisfaction seem to be mediated by the ability to effectively coordinate the care pathway, build relationships, connect to identify needs, while HCP satisfaction might be connected to the current critical points of care delivery of the current operative model, to the appropriateness of the services provided, to the degree of freedom as freelance physicians, and to the option of applying alternative management solution to human resources to improve organizational performance.

**3.1 Being satisfied or not with the care delivered by the PHP**

In general, HCPs seem to be satisfied with the experience at the PHP. The general perception is that the PHP innovative model is effective in responding to the shortage of GPs and users seem to be satisfied with the service. Additionally, users are happy with the services provided by the nursing staff at the PHP and interact with the service.

*We are in a phase where there has been and will be a severe shortage of doctors, so for them rather than being without a doctor, having no one to look after them, still having a stable figure to refer to I see them all very, very happy. (*I 8).

**3.2 Coordinating the care pathway**

HCPs emphasize the importance of accompanying users along their care pathway. This involves supporting users in tracking their progress, gathering documentation, identifying and prioritizing needs with their families, activating additional services when necessary, and involving the family network during critical situations.

*The patient of a certain age who has some problems and needs medications, to maybe tweak the therapy that is not going well, who maybe needs an extra access to control the blood pressure, users of this type basically, who need a figure able to care for them and that keeps them followed. (*I 7).

**3.3 Building relationships with users**

Users seemed to be generally happy with the care received; however, they valued the relationship with HCPs more than the quality of the service. HCPs note that even if at first there was distrust with new physicians then overtime users have given positive feedback. A positive note is that it is easier to get in touch with HCPs at the PHP compared to the local GP practices and telephone support from nurses reassures users waiting physician contact. Some users desire a consistent physician as their point of reference to build trust, although physicians note that treatment remains consistent regardless of the on-call physician.

*It’s a new thing and I often see them also saying eh but last time there was your colleague today he’s not here, so maybe they too would like to have a doctor, a familiar face, here they see them from time to time. But from the point of view, I mean, of the prescriptions of therapies there is no gross difference, here. (I 6)*

**3.4 Pointing out critical points of current operative model of care delivery**

Some issues involving the current management of the innovative model of primary care delivery have been highlighted. Among them, the presence of a high turnover, the rotation of physicians in a variety of different primary care services (nursing home, PHP, GP temporary substitution due to sickness leave), shifts managed on a weekly basis, lack of organizational resources such as a single electronic healthcare record, and the impossibility for physicians to maintain a pool of users.

*i.e. working in a big group in the sense where there are 20/30 doctors it’s difficult to have continuity, maybe one doctor makes a thought, another doctor comes and maybe he changes the therapy and so on. As it also happens in the hospital to be honest, because even there the patient is not always followed by the same doctor, who is on duty visits the patient. (I9)*

Physicians proposed alternative management solutions of human resources to improve organizational performance of PHP, improve the organization of rostering, and give more continuity to the same groups of doctors rotating in services, including nursing homes.

**3.5 Discuss the appropriateness of the services and prescriptions provided**

The current operative model of primary care delivery leads to some limitations. First, PHP physicians seem to spend less time educating users than prescribing. It is difficult to ensure prescriptive appropriateness in the absence of continuity of care and of a single health record that would enable practitioners to see the history of activities. Additionally, HCPs note a defensive medicine approach, leading to excessive tests due to uncertainty regarding result review or timing among colleagues.

*But it takes time to explain to the patient that it is not needed, and in my opinion in the activity of the* PHP *there is less inclination to do this. On chronic pathology there is a lack of tools. There are tools but there are insufficient electronic tools. I may not have control over the patient but if I have a history where I see what has been prescribed, like a medical record of the GP kept well… if I see what has been prescribed, the record of what he has done, I can say to him look you did 6 months ago the ultrasound of the neck, everything was fine, we will do it again in a year and a half, no need to do it before. There instead you sometimes try to look to see if you find [the history…], but if you don’t find whatever, you just prescribe it again. (I5)*

**3.6 Current electronic health tools and potential for new digital health interventions**

Professionals highlighted challenges due to the absence of a single electronic health system. They emphasized that utilizing one electronic management system would enhance continuity of care and streamline information transfer, in contrast to the current use of five different healthcare software systems. HCPs view telemonitoring and digital health interventions, which monitor parameters and provide warning signals, as potentially beneficial tools. However, they acknowledge that older adults may face challenges in using telemonitoring systems and suggest providing them with caregivers who can assist in using the tool. Furthermore, a data platform (such as digital intervention or telemonitoring) would be a valuable resource only if it can interface with the electronic healthcare record, rather than being another external software to open. Conversely, HCPs have questioned the utility of video calling, as patients typically find satisfaction with regular calls and are reassured by in-person visits.

*if the blood pressure monitor sends me the data directly to the chart it’s already a support for me, but they have to come in the electronic healthcare record and not in another external software that I have to open. (I 8)*

## Discussion

From the results emerge that this innovative model of primary care delivery has been effective in answering the needs of the users thanks to the flexible approach and the introduction of a interdisciplinary team led by an FCN, even though some critical aspects remain. According to HCPs, patient satisfaction appears to be influenced by aspects such as effective care pathway coordination, relationship building, and the proper identification of patient needs. Meanwhile, HCP satisfaction may be linked to critical aspects of care delivery in the current operative model, service appropriateness, freedom as freelance physicians, and the ability to implement alternative management solutions for improved organizational performance.

The introduction of a family nurse appears to be one of the key factors for the effectiveness of PHP. Some of the interviewed physicians highlighted as a criticism the fact that with the PHP model physicians might be able to answer only to the acute needs and clinical symptoms of the individual family member. On the other hand, the family nurse maintains a clear vision on the family system and on the general situation of the patient. In this regard, the effectiveness of the role of nurses with advanced training in community care compared to physician-led or usual care has been recently investigated by Htai *et al*. (Htay and Whitehead, 2021). This systematic review highlighted statistically significant positive effects in favour of nurses in relation to patient care and service outcomes, including symptom severity, physical function, waiting times, and costs. Moreover, our results reported that patients seem to value the importance of having a consistent figure of reference in charge of their clinical pathway and with whom they could build a relationship of trust, regardless of the professional profile and background. This approach is in line with the emergence and development of patient navigation around the world (Manderson *et al*., 2012), in response to the growing complexity of healthcare service delivery, the aging population, increased comorbidity, and social inequalities in population health (Gimpel *et al*., 2010; Hunter and Bengoa, 2022). It signals an important shift in the recognition that health care and social care are inextricably linked especially to address the social determinants of health (Carter *et al*., 2018). Medically complex patients in primary care experience fragmentation and gaps in service delivery and navigators assist with fragmentation of the health and social health care system through various methods including communication with multiple service providers (Palinkas *et al*., 2010; Carter *et al*., 2018).

Nevertheless, our results showed that collaboration within a team can present challenges, as proactive, interdisciplinary patient care may sometimes face obstacles due to communication difficulties between physicians and nurses. The situation can be exacerbated by frequent turnover among physicians. In this regard, evaluating the factors that support or impede interdisciplinary teamwork in primary care is crucial (O’Reilly *et al*., 2017). An overview of reviews on interdisciplinary collaboration in primary care (Rawlinson *et al*., 2021) highlighted among its facilitators co-location and recognition of other professionals’ skills and contribution, elements included by the PHP model since its inception. Among the main barriers, lack of time and training, lack of clear roles, fears relating to professional identity and poor communication. In this regard, it’s important to highlight that this care model has been introduced during the COVID-19 pandemic (Khalil-Khan and Khan, 2023), and the introduction of family nurse practitioners in primary care constitutes a novelty for the Italian Healthcare System (Marcadelli *et al*., 2019; Musio *et al*., 2022), which implies that professionals might need some time to adjust to new role dynamics in the context of a new care model.

As regarding professional satisfaction with the new care model, professionals seem to have positively accepted the change but underline practical challenges resulting from the lack of interoperability between the available IT systems and raise concerns on appropriateness of prescriptions and service delivery. This last concern could be due to a more defensive approach to prescriptions for patient safety assurance in the light of the lack of continuity of care with the same physician (Shenoy *et al*., 2022). A possible solution to this problem, as stated by the recent analysis of Kakemam *et al.*, could refer to structured training and education of all the equipe regarding prescription appropriateness (Kakemam *et al*., 2022).

Current trends show global nursing shortages, and Italy makes not exception, therefore it could be argued that this model is not sustainable due to the shortage not only of GPs, but also of nurses (Hunter and Bengoa, 2022). However, the PHP model represents an innovative and adaptable solution to address healthcare workforce shortages, leveraging interdisciplinary collaboration and advanced nursing roles. While the global shortage of nurses is a recognized challenge, the PHP model addresses this issue through several structural and operational strategies: **(**a) enhanced role of FCNs: the PHP model leverages the expertise of FCNs with advanced training to perform a broad range of functions, including proactive patient monitoring, chronic disease management, and integration with social services. This diversification of roles promotes effective task sharing among HCPs. Recognizing the challenges posed by the nursing shortage, Italy is addressing this issue through strategic investments in advanced nursing education and the creation of expanded clinical leadership roles, supported by adequate compensation. The PHP model exemplifies this forward-thinking approach by integrating FCNs leaders, positioning these roles as attractive career pathways that foster the development of a skilled nursing workforce and drive healthcare innovation. (b) Flexible and efficient care delivery: by employing a team-based approach, where nurses and physicians rotate shifts while maintaining extended service hours (8am–8pm), the PHP enhances accessibility and continuity of care. This flexibility maximizes the utilization of available resources, ensuring comprehensive service delivery despite possible workforce constraints; (c) focus on proactive medicine: the integration of proactive strategies such as targeted monitoring of vulnerable populations and therapeutic education streamlines care pathways and reduces the overall burden on acute services, benefitting the overall sustainability of healthcare services. This strategic focus amplifies the system’s efficiency, trying to alleviate pressure from staff shortages; (d) scalability and local adaptation: the model’s design allows for adjustments based on local workforce availability and population needs. This adaptability is key to its long-term sustainability, offering a replicable framework for regions facing similar challenges.

On another note, it is important to highlight that the PHP model represents a distinct model from home healthcare services. Unlike home-based nursing care, the PHP operates out of a physical centre where patients visit to receive primary care services, with the additional option of home visits, when required (Kringos *et al*., 2013). It features an interdisciplinary team of physicians, nurses, and staff collaborating to provide a broad scope of services beyond just in-home caregiving (Xyrichis and Lowton, 2008). Moreover, the PHP aims to serve the general population, not just homebound individuals, by offering accessible care through extended 8am–8pm hours. With its facility-based setting, interdisciplinary team, expanded service offerings, and availability to a wider patient population, the PHP model introduces an innovative integrated primary care approach differentiated from the more limited domain of home healthcare nursing (Bodenheimer and Pham, 2010).

## Limitations

This study has a few limitations. First, the limited sample size. On this regard, it is important to highlight that the PHP is a single centre located in a peripheral area. However, the researchers achieved data saturation. Data saturation in qualitative research occurs when no new information or themes emerge from the data (Saunders *et al*., 2018). Through an iterative process of data collection and analysis using a grounded theory approach, the researchers determined data saturation was reached as no new codes or categories were identified, indicating a comprehensive understanding of the phenomenon from the participants. Second, the study has been performed in a dynamic model of care, shaped by the contextual local necessities. For this reason, result transferability should be considered considering the need to adapt primary and community care to the needs of local communities

### Conclusions

This innovative model of primary care delivery has been deemed acceptable as an alternative way to deliver primary care compared to the traditional GP centric model of care in changing organizational dynamics. Users access the PHP with primary care needs mostly related to disease pathway coordination, referrals and medication management, with chronic and older adults demonstrating to be the populations presenting more needs that need to be addressed by the PHP. The PHP presents a structured interdisciplinary workflow, with a dynamic rotation of physicians based on availabilities and the steady presence of family nurses as a point of reference for patients, with a proactive medicine approach to monitor and manage fragile populations. Patient satisfaction seems to be mediated by the ability of HCPs to coordinate the care pathway and to build trust relationships. Health professionals’ satisfaction on the other hand depends on the opportunity to maintain a flexible work schedule and to propose alternatives to some of the critical points of care delivery currently in place at the PHP.The next steps for this research program will include investigating the perspectives and experiences of the other key user group – patients and their caregivers receiving care through the PHP model. Understanding their needs, satisfaction, and lived experiences is crucial for comprehensively evaluating this innovative approach to primary care delivery.

Implications for the profession and/or patient care.

This innovative model of care highlights the impact of FCN as a coordinator of patient care, as well as a trusted point of reference for patients. It constitutes an example of the development of the recently introduced figure of family nurse in Italy and its potential for primary care enhancement.

Impact (Addressing:)

- What problem did the study address? The study addressed the challenge of delivering primary care in the context of a generalized lack of availability of GPs, and the innovative model of care that was developed to answer to this need.
- What were the main findings? The study found that final users of the model were generally satisfied with this care delivery model, although they highlighted the necessary improvements to be implemented in the future.
- Where and on whom will the research have an impact? The research will have an impact in all those contexts looking into introducing alternative models of primary care delivery to the traditional GP model.

Reporting Method: State here that you have adhered to relevant EQUATOR guidelines and name the reporting method.

The results of the study were reported using the COREQ checklist for quality research reporting.

Patient or Public Contribution: Include a paragraph that details how patients, service users, caregivers, or members of the public were involved in your study. This may be the design or conduct of the study, analysis or interpretation of the data, or in the preparation of the manuscript.

Patients and service users were involved in the study since its inception. They were consulted in the process of study design, and they had the opportunity to review and interpret the data along with the researchers before to prepare the manuscript.

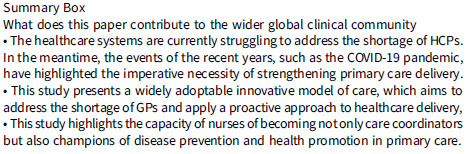



## Supporting information

Mezzalira et al. supplementary material Mezzalira et al. supplementary material

## Supplementary material

The supplementary material for this article can be found at https://doi.org/10.1017/S1463423625000210
