# Supplementary figures and images for: The experience of an innovative interdisciplinary model of primary care delivery in changing organizational dynamics: a grounded theory study
Source: Prim Health Care Res Dev. 2025 Feb 28;26:e25. doi: 10.1017/S1463423625000210 (PMC11883782; doi:10.1017/S1463423625000210)

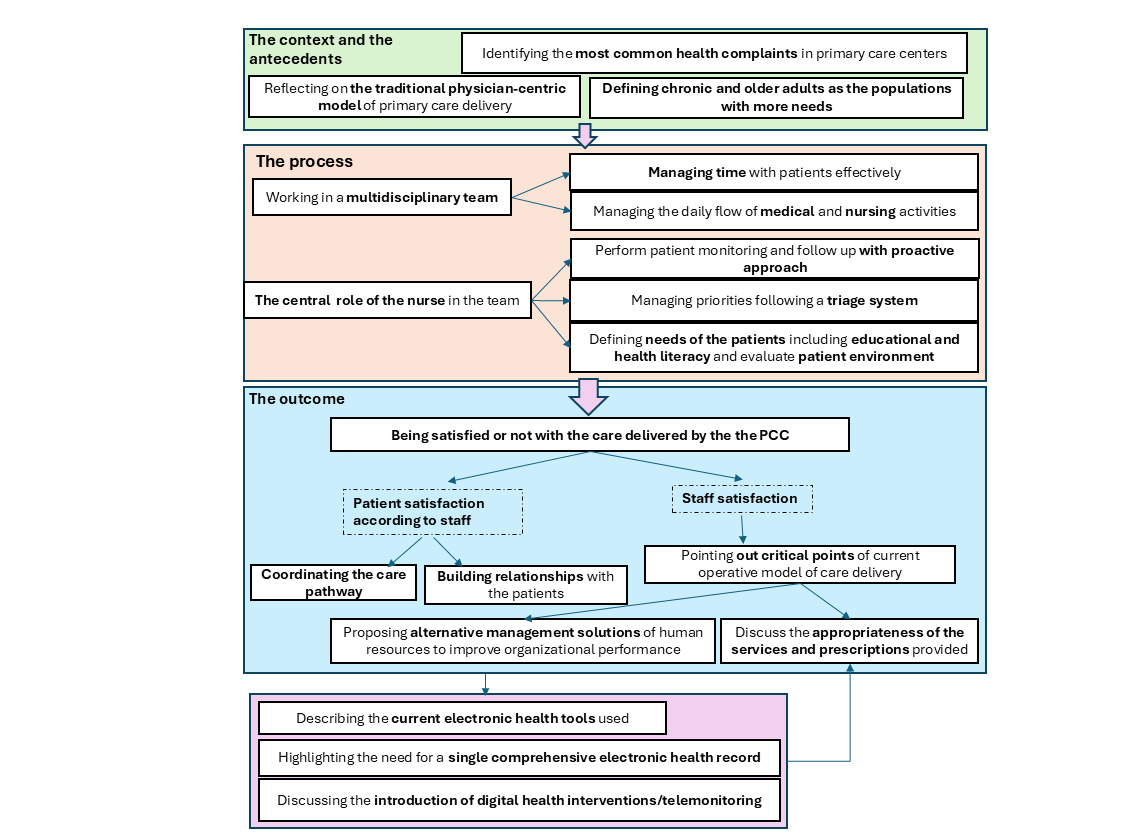

Supplement: Mezzalira et al. supplementary material [file S1463423625000210sup001.tiff]
